# A Critical Appraisal of the Social Norms Approach as an Interventional Strategy for Health-Related Behavior and Attitude Change

**DOI:** 10.3389/fpsyg.2018.02180

**Published:** 2018-11-06

**Authors:** Robert C. Dempsey, John McAlaney, Bridgette M. Bewick

**Affiliations:** ^1^Staffordshire Centre for Psychological Research and Centre for Health Psychology, Department of Psychology, School of Life Sciences and Education, Staffordshire University, Stoke-on-Trent, United Kingdom; ^2^Department of Psychology, Faculty of Science and Technology, Bournemouth University, Bournemouth, United Kingdom; ^3^Division of Psychological and Social Medicine, School of Medicine, Leeds Institute of Health Sciences, University of Leeds, Leeds, United Kingdom

**Keywords:** social norms, health behaviors, normative misperceptions, substance use, intervention

## Abstract

The Social Norms Approach is a widely used intervention strategy for promoting positive health-related behaviors. The Approach operates on the premise that individuals misperceive their peers’ behaviors and attitudes, with evidence of under- and over-estimations of behaviors and peer approval for a range of positive and negative behaviors respectively. The greater these misperceptions, the more likely an individual is to engage in negative behaviors such as consuming heavier amounts of alcohol and other substances and reduce positive behaviors such as eating healthily and using sun protection. However, there are many complexities associated with the use of social norms feedback in interventions and empirical studies. Many social norms interventions do not attempt to change misperceptions of social norms or measure changes in normative perceptions pre- and post-intervention. This has led to a conflation of generic social norms interventions with those that are explicitly testing the Approach’s assumptions that it is misperceptions of peer norms which drive behavior. The aim of the present review was to provide a critical appraisal of the use of the Social Norms Approach as an intervention strategy for health-related behaviors, identify the current issues with its evidence base, highlight key opportunities and challenges facing the approach, and make recommendations for good practice when using the approach. There are three core challenges and areas for improved practice when using the Social Norms Approach. Firstly, improvements in the methodological rigor and clarity of reporting of ‘social norms’ research, ensuring that studies are testing the approach’s assumption of the role of misperceptions on behaviors are differentiated from studies investigating other forms of ‘social norms.’ Secondly, the need for a more explicit, unified and testable theoretical model outlining the development of normative misperceptions which can be translated into interventional studies. Finally, a need for a more robust evaluation of social norms interventions in addition to randomized controlled trials, such as the inclusion of process evaluations, qualitative studies of participant experiences of social norms feedback, and alternative study designs better suited for real-world public health settings. Such improvements are required to ensure that the Social Norms Approach is adequately tested and evaluated.

## Introduction

Social Norms are important determinants of health-related behaviors and feature in a number of prominent psychological theoretical models of health behavior (Ajzen, 1991; Gerrard et al., 2008). Social norms are typically defined as “*rules and standards that are understood by members of a group, and that guide or constrain social behaviors without the force of law*” (Cialdini and Trost, 1998, p. 152), and often relate to a perceived social pressure to engage or not engage in specific behaviors (Ajzen, 1991). Social norms tend to work in an implicit manner, where individuals’ perceptions of normative behaviors are used to guide behavioral patterns and intentions, but can be based on direct and explicit communication between group members (Hogg and Reid, 2006). Although social norms can be conceptualized in different ways (Lapinski and Rimal, 2005; Chung and Rimal, 2016), the role of perceived normative peer behaviors and attitudes have emerged as key predictors of health behaviors. There is also evidence that individuals can be poor at estimating the actual behavioral and attitudinal norms of their peers and affiliated social groups (Perkins and Berkowitz, 1986; McAlaney et al., 2011). An important consequence of such social norm misconceptions, or “normative misperceptions,” is the potential engagement in unhealthy behaviors due to a false belief that such behaviors are commonplace amongst one’s peer group (McAlaney et al., 2011). Evidence that individuals often misperceive their peers’ engagement in various positive and negative health-related behaviors has led to the development of the ‘Social Norms Approach’ (SNA) as a behavior change strategy.

### The Social Norms Approach

The SNA has its origins in a study conducted by Perkins and Berkowitz (1986) which observed that university students tended to overestimate their peers’ alcohol consumption (Perkins, 2003; McAlaney et al., 2011). Subsequent research has since substantiated Perkins and Berkowitz’s (1986) findings of students’ overestimations of peers’ use of alcohol, frequency of drunkenness and attitudes toward alcohol use (Neighbors et al., 2006; McAlaney and McMahon, 2007; Arbour-Nicitopoulos et al., 2010; Boot et al., 2012; McAlaney et al., 2012, 2015). The SNA focuses on two types of (mis)perceived social norms in influencing individual behavior, these are: *injunctive norms* relating to the perceived attitudes or approval of behaviors by others; and *descriptive norms* relating to perceptions of others’ engagement in behaviors (e.g., the frequency of binge drinking) (McAlaney et al., 2010, 2011). Misperceptions of these social norms can have important implications for personal use. For example, overestimating peers’ alcohol use may make heavier consumption perceived to be a socially desirable behavior and, as predicted by social comparison theory, individuals are driven to match what they perceive to be the social norm (Festinger, 1954). Such misperceptions have been associated with a range of behaviors, including increased alcohol and other substance use, amongst others such as dietary behaviors (e.g., Martens et al., 2006; Neighbors et al., 2008a; Arbour-Nicitopoulos et al., 2010; Perkins J.M. et al., 2010). The effect of these perceived peer descriptive norms on behavior appears to be greater than the effect of behaviors on perceived norms (Neighbors et al., 2006), suggesting a causal pathway from misperceived norms to changes in behavior. There are several factors which feed such misperceptions; for example, the public behavior of a small number of individuals (e.g., students being visibly drunk), media coverage and discussion of such extreme minority behaviors, and the highlighting of such extremes in everyday conversation. Because of these factors, the scope and prevalence of negative health behaviors by the minority are exaggerated and the behaviors of the healthy majority are ignored (Perkins, 2003).

The majority of studies have focused on the role of social norms misperceptions on alcohol use, particularly student alcohol consumption on university campuses in North America (e.g., Neighbors et al., 2007; Larimer et al., 2009). Misperceptions of peer norms have since been observed for other negative health behaviors. For example, individuals tend to perceive that peers are more likely than themselves to smoke tobacco (Arbour-Nicitopoulos et al., 2010; Pischke et al., 2015), cannabis (Kilmer et al., 2006; Arbour-Nicitopoulos et al., 2010; Dempsey et al., 2016), and use other illicit drugs (Helmer et al., 2014). Overestimations of social norms have been evidenced for drinking sugar-sweetened beverages (Perkins J.M. et al., 2010; Lally et al., 2011), eating unhealthy snacks (Lally et al., 2011), using ‘smart drugs’ to improve academic performance at university (Helmer et al., 2016b), using non-medically prescribed sedatives and sleeping pills (Lehne et al., 2018), engaging in distracted driving behavior (Carter et al., 2014), risky sexual behaviors (Lewis et al., 2007; McAlaney and Jenkins, 2017), as well as overestimating the rates of sexually transmitted infections and unintended pregnancies amongst peers (Scholly et al., 2005). Misperceived social norms can influence perceptions of what is considered to be a healthy or attractive body image, such as misperceptions of peer desirability of thinness and muscularity (Grossbard et al., 2011). There is also evidence of over-estimations of injunctive norms toward tanned skin being considered healthy and attractive amongst women (Reid and Aiken, 2013), and of over- and under-estimations of peer weight norms amongst high school students being predictive of increased risk of personally being over- or under-weight respectively (Perkins J.M. et al., 2010). It appears that women are more likely to perceive that men are attracted to thinner female bodies compared to what men actually report, with these misperceptions associated with more dysfunctional eating attitudes and behaviors (Bergstrom et al., 2004).

Conversely, there is evidence that individuals perceive that their peers are less likely to engage in positive health behaviors, and have less favorable attitudes, in comparison to their own beliefs and the actual reported norms amongst their social group. This has included handwashing and infection control behaviors (Lapinski et al., 2013; Dickie et al., 2018), fruit and vegetable consumption (Lally et al., 2011), use of condoms (Scholly et al., 2005), attending HIV tests (Perkins et al., 2018), and the use of sun protection (Reid and Aiken, 2013). Fewer studies into the presence of misperceived or underestimations of social norms for positive health behaviors exist compared to studies investigating negative health behaviors, particularly alcohol use.

Alongside studies into the role of social norm misperceptions on behavior, the SNA has developed into a widely implemented behavior change technique (Perkins, 2003; McAlaney et al., 2011). Interventions based on the SNA aim to reduce negative and promote positive health behaviors by challenging these misperceptions of social norms (McAlaney et al., 2010). The SNA makes several assumptions about the influence of these social normative perceptions on behaviors, these are: (i) that perceived norms are consistently associated with behaviors; (ii) individuals tend to misperceive or under/overestimate their peers’ behaviors and attitudes; (iii) that such misperceptions are associated with the increased/decreased engagement in those behaviors; and (iv) interventions which correct such misperceptions should promote more positive behaviors (Perkins, 2003; Perkins H.W. et al., 2010).

Whilst many studies report the use of social normative feedback as a one of a number of intervention components which aim to promote health behaviors (e.g., Kypri et al., 2004, 2009; Saitz et al., 2007), it is often unclear whether these studies are testing the assumptions of the SNA. Many studies do not specify whether the existence of misperceptions of peer behaviors and attitudes was ascertained during the development of their ‘social norms’ interventions. Other studies also fail to test how these misperceptions influence personal behaviors and/or attitudes, and many fail to assess changes in normative perceptions over the course of the intervention as a possible treatment mechanism (e.g., as a mediator of the effect of an intervention on personal behaviors and attitudes), in line with the central tenets of the SNA. These concerns may limit the evaluation of the SNA in terms of its effectiveness as a health-related behavior and attitude change technique by ignoring its fundamental assumptions regarding the role of normative misperceptions on outcomes.

### The Present Review

Whilst the SNA has demonstrated promise as a behavior change intervention, with the potential for its cost-effective use with large groups of participants, there is a lack of critical commentary regarding the application of the SNA and the use of ‘social norms’ feedback more broadly. Therefore, the aims of this review are to: (i) critically appraise the use of the SNA as a behavior and attitude change intervention for a range of health-related behaviors and evaluate the evidence base for the approach; (ii) identify the current challenges and opportunities associated with using the SNA as a behavior and attitudinal change technique; and (iii) identify good practice when using the SNA in future health-related behavior change interventions.

## The Social Norms Approach as a Behavior Change Intervention

### Assumptions of the Approach

The SNA’s primary assumption is that misperceptions of social norms drive the engagement in negative health behaviors, but such behaviors can be mitigated by challenging these misperceptions through informational feedback about actual reported norms (Perkins, 1997, 2003; McAlaney et al., 2011). SNA interventions differ from fear-based, ‘health terrorism,’ approaches which feature scare tactics or highlight the adverse possible consequences of negative behaviors in order to promote behavior change (Scholly et al., 2005; McAlaney et al., 2011). Such fear-provoking appeals are considered to facilitate behavior change by emotionally confronting individuals with the consequences of their behavioral choices (Kok et al., 2018), however, the effectiveness of these strategies in health promotion has been disputed (for a debate, see: Malouff, 2018; White and Albarracin, 2018). Some criticisms of fear-based approaches include the potential promotion of high-risk behaviors as being more commonplace than the reality, as well as highlighting the extreme and unlikely outcomes of behaviors which could be dismissed for their lack of realism (McAlaney et al., 2011).

In contrast to ‘health terrorism’ approaches, the SNA presents feedback which highlights actual positive reported behaviors and/or attitudes amongst a target group, using subtle social influence pressure to promote conformity to healthier, actual norms (Perkins, 2003; McAlaney et al., 2010). Good practice when designing SNA interventions includes the active involvement of the target participants in data collection and the design of intervention materials (McAlaney et al., 2010). Indeed, SNA interventions should base feedback on the actual reported behaviors and attitudes from the same or similar group as the target population to ensure that such feedback is perceived to be credible, relevant, and representative of the target group’s norms (Perkins, 2003; McAlaney et al., 2010). However, not all ‘social norms’ interventions directly base their feedback on survey data from the target group (e.g., Kulik et al., 2008), which may undermine the perceived realism, persuasiveness and ultimately the effectiveness of normative feedback. Referent groups featured in SNA interventions vary but typically include peers who share some group membership (e.g., university students studying at the same campus; Neighbors et al., 2010b; LaBrie et al., 2013), including individuals living in the same community, working at the same employer, or using a shared public location (e.g., Perkins H.W. et al., 2010; Bewick et al., 2013; Perkins et al., 2018). SNA feedback should be presented as coming from the wider social group associated with the target population, and not be perceived to come from an authority figure, to avoid changes in behavior and attitude due to obedience pressure or fear. SNA-informed interventions targeting misperceptions of social norms have tended to focus on substance use, most commonly alcohol (Neighbors et al., 2010b, 2012; LaBrie et al., 2013), with others now targeting cannabis (Lee et al., 2010, 2013), polysubstance (Pischke et al., 2012; Helmer et al., 2016a) and tobacco use (Hancock et al., 2002; Zhang et al., 2010; Elsey et al., 2015). It is notable that fewer SNA interventional studies have targeted non-substance related behaviors, although SNA interventions focused on improving sun protection (Reid and Aiken, 2013), handwashing (Lapinski et al., 2013), safe sexual behaviors (Scholly et al., 2005) and other risky behaviors (e.g., drink-driving, Perkins H.W. et al., 2010) have been published.

### The Use of Descriptive Versus Injunctive Norms Feedback in SNA Interventions

Most SNA interventions have tended to focus on the role of descriptive norms in changing behaviors by highlighting the difference between the perceived versus the actual reported prevalence of behaviors (e.g., Lee et al., 2010; Neighbors et al., 2010b; LaBrie et al., 2013). A small number of injunctive norms focused interventions have been conducted which instead highlight discrepancies between perceived and actual reported peer approval (e.g., Prince and Carey, 2010; Reid and Aiken, 2013). To our knowledge, no studies to date have directly compared the effectiveness of descriptive versus injunctive norms feedback on the same health behavior. Instead, research has focused on the effects of either descriptive or injunctive norms feedback on outcomes, with a few studies investigating the effect of combining descriptive and injunctive feedback. Although, an older meta-analysis suggested that greater misperceptions (or ‘self-other discrepancies’) are found for injunctive compared to descriptive norms for student alcohol use (Borsari and Carey, 2003), suggesting the potential utility of SNA feedback in reducing misperceptions of the perceived peer approval of behaviors. There are some studies which have included both descriptive and injunctive norm feedback messages in the same intervention (e.g., Ridout and Campbell, 2014), however, it is difficult to elucidate whether it is the descriptive or injunctive feedback which is the more effective on facilitating behavior or attitudinal change in such studies.

A number of studies have reported significant reductions in drinking behaviors (e.g., Neighbors et al., 2009, 2010b) and perceived peer drinking norms post-SNA descriptive normative feedback (e.g., Neighbors et al., 2010b; Lewis et al., 2014). Others have report an increased likelihood of remaining abstinent from alcohol amongst non-drinkers following descriptive normative feedback (Larimer et al., 2007). In terms of other substances, reductions in student cannabis use (i.e., joints smoked per week) were reported at a 3-month follow-up after a SNA cannabis use intervention (Lee et al., 2013). However, an earlier web-based SNA intervention did not find an overall effect of viewing cannabis-use descriptive norms feedback on cannabis use behaviors compared to a control, but reductions in cannabis use amongst a subgroup of students with a family history of drug use was noted at a 6-month follow-up (Lee et al., 2010). Beyond substance use, reductions in money lost and problems associated with gambling, and more accurate perceptions of peers’ money won and lost through gambling, were observed amongst student gamblers following descriptive norms SNA feedback (Neighbors et al., 2015). In terms of the effectiveness of injunctive norms feedback, greater reductions in students’ perceived peer drinking rates and peer approval have been reported after a brief injunctive norms intervention compared to a control (Prince and Carey, 2010). Similarly, improvements in perceived peer sun protection injunctive norms, intentions and behaviors amongst a female community sample were reported following an injunctive norms SNA feedback intervention (Reid and Aiken, 2013). These are a small number of example studies which have reported positive outcomes following SNA feedback, however, as discussed earlier, notably fewer SNA interventions have provided feedback on injunctive norms alone compared to the number of studies which include descriptive norms feedback.

Given the relative scarcity of research into the effects of viewing injunctive norm-based feedback on behaviors, it is a little unclear if injunctive SNA interventions are more effective for changing personal approval of behaviors or changing behaviors compared to descriptive norms feedback. It is also unclear how changing perceived injunctive norms may interact with perceived descriptive norms when facilitating behavior change. It may be that misperceptions of peer descriptive norms are also accompanied with an implicit misperception of peer approval of the behavior and, vice versa, perceiving that most of your peers approve of a specific behavior may feed misperceptions of how often such behaviors are enacted. Although, there is some empirical evidence for the interacting role of perceived descriptive and injunctive norms on personal behavior. For example, one study reported stronger associations between perceived peer drinking norms and personal alcohol consumption amongst students who thought their peers were more approving of alcohol use (i.e., had more permissive perceived injunctive norms), especially those students who drank for social reasons (Lee et al., 2007). The relationship between perceived descriptive and injunctive norms may depend on the health behavior under investigation. For example, one study reported that self-efficacy mediated the relationship between descriptive norms, but not injunctive norms, with cannabis-use outcomes despite a positive correlation between injunctive and descriptive norms (Walker et al., 2011). Other studies have suggested that perceived descriptive and injunctive norms have independent effects on behavior (e.g., cannabis use, Neighbors et al., 2008a); however, many of these studies are cross-sectional in nature making it difficult to conclude the temporal order of the relationship between perceived descriptive and injunctive norms on behaviors. There are some challenges remaining for the SNA, including the question of whether injunctive or descriptive normative feedback is more effective in changing behaviors, whether there is an interaction between perceived injunctive and descriptive norms in facilitating changes in behaviors and/or attitudes (and what form this relationship may take), and whether this is related to the specific behaviors, participant group and/or social context under scrutiny.

### Delivery of Social Norms Feedback in SNA Interventions: Print and Mass Media Campaigns

Early SNA interventions took the form of social marketing campaigns targeted at the group level, with the use of mass media to disseminate social normative feedback often via print-based adverts and posters (Scholly et al., 2005; Lewis and Neighbors, 2006; Turner et al., 2008; Perkins H.W. et al., 2010; McAlaney et al., 2011). There is some support for the effectiveness of SNA marketing approaches for changing behaviors and misperceived norms (Lewis and Neighbors, 2006). For example, reductions in misperceptions of the rates of drink-driving, in the percentage of young adults who drove in the hour after consuming 1–2 alcoholic drinks, and increases in the use of designated drivers were reported after an intensive, 15 month long community-based SNA media campaign in Montana, United States, which included radio and television advertisements (Perkins H.W. et al., 2010). However, a 9-month sexual health SNA campaign which presented normative feedback to students via on-campus posters, as well as via give-away pens and sweets, failed to observe any significant changes in sexual health behaviors (e.g., condom use) and perceived sexual health behaviors pre-to-post intervention (Scholly et al., 2005). Scholly et al. (2005) discuss a range of challenges encountered during their campaign, including the withdrawal of two sites due to the perceived offensive and explicit nature of the posters by campus staff. In addition, issues with on-campus resources and staffing, specifically the ability to co-ordinate a mass media campaign which saturates the campus with normative feedback messages, appeared to limit the effectiveness of their intervention (Scholly et al., 2005). There was also a lack of a control comparison group in this study and the focus on protective sexual health behaviors (i.e., condom use) may not have accounted for some students at the target sites choosing not to be sexually active, which may explain the lack of significant changes in condom use and related behaviors noted in the study (Scholly et al., 2005). It may be that exposure to the SNA intervention in this study protected against time-related rises in rates of unprotected sex but without an adequate no-intervention comparison group this is uncertain. In contrast, a 6-year long poster and print-based media SNA campaign focusing on reducing student alcohol use reported significant decreases in the odds of students experiencing negative alcohol-related consequences (e.g., drink driving, risky sexual behaviors) alongside high rates of intervention exposure and recollection of normative messages amongst target participants (Turner et al., 2008). Using printed media to deliver SNA interventions is associated with challenges in the ability to target and expose participants to feedback, as well as in ensuring target groups view and attend to messages, in addition to the physical and resource-related challenges associated with saturating targeted social settings with feedback messages. Studies using a mass-media approach have also tended to deliver interventions over many months or years, which may be accompanied with significant costs in terms of resources and finances.

Although print-based campaigns can easily target large groups of participants, they are limited to presenting actual reported social norms and cannot provide tailored feedback explicitly comparing individuals’ own behaviors with perceived and actual reported peer behaviors. SNA interventions targeted at the group level may also be limited to assessing group-level changes in perceived norms, attitudes and behaviors, and unable to account for individual-level changes in these variables (e.g., Perkins H.W. et al., 2010). It may also be difficult to track the exact exposure of SNA feedback in mass media campaigns, especially in terms of individual exposure to and processing of feedback (Lewis and Neighbors, 2006), with some prior studies relying on participant self-reports to estimate the exposure to and recollection of normative feedback (Perkins H.W. et al., 2010). The exposure to and engagement in SNA feedback could be an important moderator of the effectiveness of SNA campaigns on changing behavior, attitudes and perceived norms. A further limitation of mass media campaigns is the potential that social normative feedback is time-limited in its appeal to target groups; whether participants continue to attend to SNA media presented over time, once such messaging is no longer novel, is unclear. However, some innovative approaches to engaging target groups in SNA media campaigns have been reported (for examples see Bewick et al., 2013).

### Delivery of Social Norms Feedback in SNA Interventions: Computerized Personalized Feedback

The increasing sophistication of computer technology has afforded the development of increasingly personalized social normative feedback interventions, which may account for the shift toward more computerized interventions in the SNA literature. A number of web-based SNA interventions have been conducted, with reports of reductions in substance use behaviors (e.g., Lee et al., 2013; Collins et al., 2014), perceived norms related to substance use and/or risky sexual behaviors (e.g., Lewis et al., 2014; Patrick et al., 2014; Ridout and Campbell, 2014), as well as the experience of problems associated with substance use (e.g., Doumas et al., 2011a; LaBrie et al., 2013), after exposure to web-based personalized SNA feedback. Of particular note across the web-based interventions published to date has been the relatively time-limited effects of normative feedback on outcomes, with some studies failing to observe treatment effects at a 6-month follow-up (e.g., Neighbors et al., 2010b; Lee et al., 2013). Many web-based SNA interventions are brief in nature and often consist of a small number of feedback messages, which may not be effective for eliciting longer-term changes in behaviors and may need additional ‘top up’ delivery to facilitate changes in perceived norms, attitudes and behaviors (Neighbors et al., 2010b).

An additional benefit of personalized SNA interventions is the ability to target specific subgroups of individuals, with studies delivering SNA feedback to specific student groups such as abstinent and light alcohol drinkers (e.g., Neighbors et al., 2011), heavy drinkers (e.g., Lewis et al., 2014; Ridout and Campbell, 2014), student athletes (e.g., Doumas et al., 2010) and those who had violated campus alcohol-control policies (e.g., Doumas et al., 2011a,b). Whilst these interventions have primarily focused on students, it is feasible that other non-student social groups could be targeted in these studies (e.g., specific subgroups of general population or community samples). These personalized SNA interventions have also featured feedback tailored to the individual’s demographic characteristics (e.g., age, sex, membership of student clubs and societies; LaBrie et al., 2013) and to event-specific behaviors (e.g., drinking during birthday celebrations and ‘Spring Break’; Neighbors et al., 2012; Lee et al., 2014). An in-depth discussion of the tailoring of SNA feedback to specific referent groups will be discussed in the next section.

The ability to deliver more tailored personalized interventions makes it easier to present participants with more salient and detailed feedback on their own behaviors and/or attitudes, their perceptions of their peer behaviors/attitudes, and finally the actual reported norms from their peer group (Neighbors et al., 2004; Lewis and Neighbors, 2006; McAlaney et al., 2010). This tripartite approach should clearly highlight discrepancies between individuals’ perceived norms and the actual reported group norms (Lewis and Neighbors, 2007), something which is difficult to accomplish with print-based campaigns. In addition, some of the issues encountered with print-based SNA campaigns, such as ensuring that target participants fully attend to feedback, could remain with computerized interventions. For example, participants could easily navigate away from webpages or simply be distracted by their immediate environment, but this may be representative of real-world experiences of engaging with online normative feedback (Neighbors et al., 2012).

## Critical Appraisal of the Current State of the Social Norms Approach Literature

An earlier review and commentary on the development of the Social Norms Approach identified a number of actions for the field to address (McAlaney et al., 2011). The first two actions related to better understanding the role of group identification on the development of normative misperceptions, and how individuals understand and visualize their normative referent groups included in feedback (McAlaney et al., 2011). The third action was the need for a more nuanced understanding of how new and developing technologies can facilitate the dissemination of social normative feedback (e.g., mobile applications and social media) (McAlaney et al., 2011).

### The Influence of Group Identification on Social Norms Misperceptions and Intervention Effectiveness

Group identification is of key importance in explaining the influence of normative misperceptions on behavior, especially when considering which normative messages hold the most relevance and persuasive power for the target population. From a theoretical perspective, social identity approaches highlight the role of self-categorization in the compliance to group norms (Hogg and Reid, 2006). Broadly speaking, individuals who identify more closely with a given social group are more likely to adhere to the norms of that group, partly as a need to fit in with the group but also as a guide for one’s own behavior (Hogg and Reid, 2006; Hornsey, 2008). The use of more proximal and salient referent groups in studies should increase the influence of perceived social norms on behaviors by making norms more relevant to the individual and should be more effective in eliciting behavior and attitude change.

Several studies have investigated the role of social identification on the misperceived norms-behavior relationship and the effectiveness of different levels of personalized feedback on behaviors. There is some evidence that the more specific the referent group, e.g., in terms of students’ identification with other same-sex and same-ethnicity peers amongst other characteristics, the stronger the association between perceived group norms and personal drinking behaviors (Lewis and Neighbors, 2004; Larimer et al., 2009; Neighbors et al., 2010a). More personalized feedback messages for alcohol use have been rated as more interesting and potentially more impactful amongst student populations compared to more generic, mass media marketing messages on social norms and binge drinking (Pilling and Brannon, 2007). SNA feedback tailored to individual characteristics and group affiliation appears to be broadly acceptable to participants.

Other research has identified the moderating role of social identity and social comparison on the relationship between perceived peer drinking norms and alcohol use amongst university students (Neighbors et al., 2010a; Rinker and Neighbors, 2014). For example, stronger associations between perceived peer drinking norms with alcohol-related consequences has been reported for students with strong tendencies to compare themselves with their peers (i.e., those high in social comparison; Litt et al., 2012a), and with increased risky alcohol-use cognitions amongst those with a greater ‘need to belong’ (Litt et al., 2012b). In terms of specific referent groups, US students who more strongly identified with same-sex, same-race or same (Greek) society students, the stronger the association was between these perceived norms and alcohol consumption (Neighbors et al., 2010a). In terms of more specific facets of social identity, the increased importance of and commitment to the wider social group by individual students, and lower deference to University leadership and rules, the heavier students’ personal alcohol use (Rinker and Neighbors, 2014). Other studies have reported that perceptions of Greek fraternity and sorority drinking norms are associated with increased alcohol consumption, particularly the role of perceived injunctive norms on personal alcohol consumption and alcohol-related problems at a 1-year follow-up (Larimer et al., 2010). Other research has suggested a more complex relationship between injunctive norms and behavior; one study reported that the perceived injunctive norms of more distal reference groups (e.g., typical student peers) were negatively associated with student alcohol consumption, whilst more proximal injunctive norms (of friends and parents) were positively associated with alcohol use (Neighbors et al., 2008b). The role of social identification and the effectiveness of normative referent groups on outcomes does seem to vary according to the social context and the health-related behaviors under scrutiny. For example, one study reported that cannabis-using students identified more strongly with typical students rather than other cannabis-using students, but the associations between perceived norms and personal use were stronger amongst those who identified with other cannabis users (Neighbors et al., 2013). Empirical research suggests that more proximal referent groups, as well as stronger and closer social identification with the referent group, strengthens the association between perceived norms and engagement in substance use behaviors. However, most studies on the role of social identity in relation to normative misperceptions have tended to focus on student alcohol and cannabis use. Whether similar relationships between social identity, perceived norms and behavior exist amongst non-student groups and non-substance use behaviors is unclear.

Generally, SNA interventions have focused on university students, with the reference groups used in interventions reflecting university social groups, such as same-sex students, students in the same year of study or in same halls of residence. There are mixed findings regarding the effectiveness of more specific referent groups in SNA feedback. A number of studies have suggested that gender-specific SNA feedback has stronger effects on alcohol use behaviors for female compared to male students (Lewis et al., 2007; Neighbors et al., 2010b), including women with stronger (female) gender identities (Lewis and Neighbors, 2007). One study failed to observe significant effects of gender-specific or neutral SNA feedback on student alcohol use, but did report significant reductions in perceived peer drinking norms irrespective of whether feedback was tailored to gender or not (Lojewski et al., 2010). In a study comparing between eight different SNA interventions for student alcohol use, which varied by the degree of personalization of the normative feedback, the most generic ‘typical student’ feedback was the most effective on alcohol use outcomes compared to feedback tailored to students’ sex, race and/or university society (‘Greek’) affiliation (LaBrie et al., 2013). LaBrie et al. (2013) suggested that the typical student norms feedback allowed individuals to project their own salient characteristics on to the feedback, a process which was limited when presented with more specific referent groups. However, LaBrie et al. (2013) did not measure the participants’ identification with the delivered normative feedback, and so the lack of effect associated with more tailored feedback may reflect a weak social identification with the featured referent groups. In sum, there is mixed evidence regarding the effectiveness of more specific referent groups on behaviors in SNA-based interventions. It is somewhat unclear whether more personalized or generic SNA feedback has the greatest effects on eliciting health-related behavior change and whether this depends on the specific behavior under investigation. There is a need for further studies to compare the effectiveness of generic versus more specific SNA feedback on behavior, better understand how participants identify their salient referent groups, and understand how normative misperceptions develop in relation to social identification over time.

### The Role of New and Developing Technologies in Delivering Social Normative Feedback

The last action highlighted in the prior review was a need for a better understanding of how new technological developments may improve the dissemination of social normative feedback, such as social media and mobile technologies (McAlaney et al., 2011). Only one study has reported using social media to deliver an SNA intervention, with Facebook messages used to deliver feedback comparing actual and perceived injunctive and descriptive alcohol use norms amongst a group of university students (Ridout and Campbell, 2014). Social media has been noted as a source of information as to what the perceived social norms of a group are (Fournier et al., 2013), therefore it is important to understand the role of social media and other new technologies in the creation and dissemination of normative misperceptions. For example, Ridout and Campbell (2014) demonstrate the how the pervasiveness of social media can be used to implement the SNA, with reductions in drinking frequencies and quantities noted for participants receiving feedback via Facebook compared to a control. These innovations are important to consider given that students and young people are increasingly moving away from using forms of electronic communication such as email toward social networking media (Judd, 2010).

## Measuring Behaviors in Social Norms Interventions

### Reliance on Self-Report Measures of Behavior, Perceived Norms and Related Constructs

SNA studies and interventions have used a range of assessments of health behavior, attitudes and perceived social norms, but have predominantly used participant self-report measures. These include validated self-report measures like the Drinking Norms Rating Form (Baer et al., 1991) to measure perceived norms (e.g., Neighbors et al., 2004), the Daily Drinking Questionnaire (Collins et al., 1985) to measure alcohol use (e.g., Neighbors et al., 2004) which has also been adapted for other substance use behaviors (e.g., cannabis, Lee et al., 2013), alongside the use of other validated measures of related constructs (e.g., substance use related problems, Neighbors et al., 2010b). Other SNA studies have developed novel surveys based on existing measures of health behaviors (e.g., handwashing, Dickie et al., 2018) and recommendations for theory-based survey item wording such as those based on the Theory of Planned Behavior (e.g., Lally et al., 2011). Adaptations of existing validated measures of health behaviors and social norms have also been used to allow for cross-cultural studies and comparisons (e.g., Pischke et al., 2012).

There has, however, been an ongoing debate about the effectiveness of self-report assessments of behavior in the social norms literature, especially assessing normative misperceptions of substance use amongst university student groups (Perkins, 2012; Melson et al., 2016). Particular discussion has focused on the potential under-reporting of negative health behaviors through the use of self-report measures, the potential benefits of using objective assessments of behaviors (e.g., using breathalyzers to assess alcohol consumption) and potential order effects of assessing personal behaviors and attitudes before perceived norms (Melson et al., 2016). However, studies have failed to find such order-effects associated with the presentation of personal versus perceived norm assessments (e.g., Baer et al., 1991) [for a brief discussion of these issues, please see Perkins (2012) commentary].

### Alternative Means of Assessing Behaviors, Perceived Norms and Related Constructs in SNA Studies

Few SNA studies to date have reported using non-self-report measures of behavior and related variables, such as rater-made or objective assessments. A novel field experiment used observer ratings of male students’ handwashing behaviors after using on-campus toilets, where posters containing feedback about normative rates of handwashing were displayed in the toilets (Lapinski et al., 2013). Higher handwashing frequency, quality and attitudes were observed following exposure to social norms messages versus a no-message control (Lapinski et al., 2013). However, due to the nature of the study as a field experiment, no pre-intervention data about participants’ handwashing behaviors, norms or intentions were collected, so it is unclear if there were baseline differences in these variables or whether there were significant changes pre-to-post intervention. Another notable study used breathalyzers to assess university students’ Blood Alcohol Content (BAC) scores on return to their halls of residence after night-time drinking (Thombs et al., 2007), with students receiving their personal BAC scores the following morning as well as normative feedback on their peers’ BACs in the study’s intervention group. Contrary to predictions, Thombs et al. (2007) reported higher BAC scores in the intervention halls which was attributed to ‘rebellious drinking’ amongst a small number of students in the intervention group, who appeared to increase their alcohol consumption as part of a competition to post the highest BACs. Whilst objective assessments of behaviors have the potential to gain more reliable data compared to self-reported behaviors, ‘effective’ social norms feedback has the potential to be ‘effective’ in producing the undesired change in behavior. It should also be noted that a number of SNA studies have calculated estimates of BACs based on self-reported alcohol intake, which has been featured in some feedback messages (Turner et al., 2008; Neighbors et al., 2012). However, it is not wholly clear how well participants understand their BAC scores or whether BAC feedback is effective in changing behavior compared to feedback on normative rates of alcohol use (e.g., quantity or frequency of consumption). The use of objective assessments in SNA studies is still limited and there remains scope for further exploration of the utility of such assessments in future SNA interventions.

### Summary: Using Self-Report Assessments in SNA Studies and Future Directions

In relation to self-report assessments of behavior, it should be noted that the limitations associated with using self-reports go beyond the SNA field and apply to many areas of health and social psychology research. If used properly, self-report assessments can be a reliable source of data (Del Boca and Darkes, 2003). Nevertheless, given that the SNA is based upon presenting actual normative rates of behavior and attitudes to the target population, it is particularly important that the data being cited is seen to be reliable and as originating from the target group. This issue may in part be addressed in the movement toward the use of smartphones and other devices that quantify behavior (Ernsting et al., 2017), whether through objective assessments based on smartphone usage or through more moment-to-moment experience sampling. There is a need for further discussion and research regarding the most effective means of collecting reliable, accurate data on behaviors, attitudes and perceived social norms, to appropriately inform SNA interventions which are effective in promoting positive behavior change.

## Evaluating the Effectiveness of Social Norms Interventions

There are a number of issues associated with the evaluation of SNA interventions, including the broad evaluation of the effectiveness of the approach and of individual interventions.

### Reviews of the Effectiveness of SNA Interventions

The potential of ‘social norms’ feedback and the SNA as an effective means of behavior change has been subjected to a number of systematic reviews. For example, two Cochrane reviews on the use of ‘social norms’ interventions (broadly defined) to reduce problematic alcohol use amongst university students reported mixed-to-negative findings of the effectiveness of ‘social norms’ feedback on alcohol consumption (Moreira et al., 2009; Foxcroft et al., 2015). However, both reviews implicitly assumed that all reviewed interventions that included ‘social norms’ feedback were tests of the SNA and that all ‘social norms’ feedback aims to change normative misperceptions. Foxcroft et al. (2015) acknowledged the heterogeneity of the social norms interventions that were included in their review but stopped short of exploring the implications of this heterogeneity and implications for quality assurance and intervention fidelity. Several studies included in these reviews featured the presentation of ‘social norms feedback’ alongside other non-normative feedback, including messaging on the relationship between alcohol use and depression (Geisner et al., 2007), health authority recommendations for alcohol use (Kypri and McAnally, 2005), health risk status (Kypri et al., 2004, 2009, 2014), monetary expenditure on alcohol (Henslee and Correia, 2009; Palfai et al., 2011), alcohol-related calorie intake (Doumas et al., 2011b; Palfai et al., 2011), and negative consequences of alcohol use (Henslee and Correia, 2009). There is a risk that some of these non-normative feedback components may detract from the positive nature of social norms feedback by including fear-based messages, risk assessments and official intake recommendations made by health authorities. The use of multiple components in such studies also makes it difficult to tease apart which intervention component was effective in changing behavior, attitudes and/or perceived social norms.

Both reviews (Moreira et al., 2009; Foxcroft et al., 2015) have been systematic reviews of published randomized control trials (RCTs). RCTs encapsulate a small proportion of population-based social norms interventions. RCTs, in general, may have limited applicability to the less controlled, real-world public health settings where it is difficult to control for various confounding variables (Glasgow et al., 1999). There are other study designs which are seen as more suited to population based interventions, such as cohort studies and other observational methods (Black, 1996), and stepped wedge cluster randomized designs (Hemming et al., 2015). While such approaches may seek to ask different questions, they provide useful insights into the implementation and effectiveness of population-based interventions. There is a need for a broader systematic search and evaluation of the evidence for the SNA that enables the inclusion of studies that have employed designs better suited to testing public health interventions.

### Challenges Associated With Evaluating the Effectiveness of SNA Feedback

A more thorough evaluation of whether SNA-based feedback is effective in changing health-related behaviors is needed, whether as a standalone intervention or as part of multicomponent interventions. There is also a need to ensure that when social normative feedback is combined with other behavior change techniques this does not undermine the correction of normative misperceptions. The inclusion of non-normative feedback which highlight the risks of certain behaviors may compromise the hypothesized treatment mechanism at the heart of the SNA. At present, there is insufficient evidence about the ability of SNA-consistent feedback to produce positive behavior and attitude change when directly compared to other forms of feedback, such as non-normative information and fear-based messaging. There remains a lack of clarity about which combinations of behavior change strategies should be included with SNA-consistent normative feedback to best promote behavior change, and how this may differ according to the target behaviors and populations under investigation. As Moreira et al. (2009) acknowledged, it is difficult to separate out the treatment effects of social normative feedback from other behavior change strategies in multicomponent interventions. It is premature to discount the potential effectiveness of SNA-consistent feedback without a more thorough and robust evaluation of the SNA in comparison to other behavior change strategies and the additive effect of including SNA feedback with other like-minded strategies.

### Evaluating User Experiences of Engaging With SNA Feedback

Whilst there is a need for a more robust and thorough evaluation of the effectiveness of SNA feedback in eliciting behavior change, there is also a need to better understand participants’ experiences of SNA-consistent feedback. This includes experiences of engaging in and using computer-based personalized SNA interventions, understanding how participants comprehend and react to when viewing live SNA normative feedback, and how this effects their perceived social norms and behavior. Some novel work using the Think Aloud Verbal Protocol reported that students viewing an online social norms alcohol intervention made repeated comparisons between their feedback with their own perceptions and experiences using alcohol, and expressed some disbelief and surprise when initially viewing their feedback (Marley et al., 2016). This initial surprise may then require further reappraisal and reflection before shifts in perceived norms and behavior are achieved (Marley et al., 2016). Whilst this is only one study, it may be that SNA-based feedback works best when participants are afforded time to reflect on their current behaviors and perceptions, before (re)considering their future behaviors.

Where participant evaluations of SNA-based feedback have been reported they suggest university students generally rate social norms feedback as being convincing and positively impactful on their alcohol use. For example, US students viewing SNA feedback tailored to their intended 21st birthday alcohol use reported that their feedback was of interest, was perceived to be accurate in content and was surprising by nature (Neighbors et al., 2009). Students who were more surprised by their feedback also had lower estimated blood alcohol levels on their actual 21st birthday (Neighbors et al., 2009). There have also been informal reports that heavy-drinking students were surprised by their SNA-based normative feedback (e.g., Lewis and Neighbors, 2007). Compared with viewing an event-specific web-based SNA intervention for 21st birthday drinking intentions, students reported higher satisfaction when receiving in-person SNA feedback with a counselor (Neighbors et al., 2012). There were more positive outcomes associated with this counselor-supported intervention over web-based forms of the same intervention, which could be indicative of the Hawthorne effect (where changes in participant behavior arise from an awareness of being observed, for a review of the Hawthorne effect see McCambridge et al., 2014). Another study reported students’ evaluations of SNA feedback as being less comfortable and acceptable compared to a non-normative control intervention (Collins et al., 2014). A common feature across these studies is the role of SNA feedback in challenging held beliefs and perceptions, a process which may uncomfortable, surprising and possibly difficult to experience on initial exposure. These feelings may be easier to process when viewing SNA feedback with support of a counselor, as per Neighbors et al.’s (2012) study, compared to viewing web-based feedback alone.

In sum, evidence to date suggests that alcohol-focused SNA feedback appears to be broadly acceptable to university student participants, although how SNA feedback is evaluated by non-university students (e.g., general population samples) and in relation to other health behaviors remains unclear. That participants find normative feedback ‘surprising’ appears to be part of the mechanism for positive behavior change after engaging with SNA feedback. However, the exact nature of this possible surprise and reappraisal process in changing behavior, attitudes and perceived norms, is not fully clear and requires further investigation.

## Challenges and Opportunities Associated With Using the Social Norms Approach

There are several immediate challenges and opportunities for the advancement of the SNA. The SNA remains highly focused on student behaviors and alcohol use despite its potential applicability to a wide variety of health behaviors and settings, particularly protective and more positive health behaviors. There is the potential for the approach to be applied to understand other behaviors, such as self-harm and suicidality (Quigley et al., 2017), gambling (Larimer and Neighbors, 2003), students’ study habits (Anthenien et al., 2018), as well as addressing bullying and victimization amongst schoolchildren (Perkins et al., 2011), and intimate partner violence (Neighbors et al., 2010c). Few studies have used the SNA in non-university settings, although this is starting to be addressed by research in high schools (e.g., Elsey et al., 2015), with armed forces personnel (Neighbors et al., 2014), in community and hospital-based public health campaigns (e.g., Perkins H.W. et al., 2010; Bewick et al., 2013; Reid and Aiken, 2013), and with non-Western communities, such as novel work investigating HIV testing uptake in Uganda (Perkins et al., 2018). The potential universality of the SNA in understanding the engagement in risky behaviors, and to intervene to prevent harm across a range of settings, remains one of the approach’s key strengths. Further evaluation of the SNA outside of student samples and university settings is particularly needed.

### The Need for a More Unified Theory of How Social Normative Misperceptions Develop

Broad theoretical assumptions about the role of normative misperceptions in promoting negative and unhealthy behaviors exist in the literature. There are numerous references to ‘Social Norms Theory’ (e.g., Scholly et al., 2005; Bergstrom and Neighbors, 2006; Martens et al., 2006) but there is no one unified theoretical model which is universally applied to all SNA research. There have been attempts to outline the interactions between normative perceptions, attitudes and behaviors, and link these to potential interventional approaches (Perkins, 1997; Rimal and Real, 2005), which provides a foundation upon which a unified comprehensive theoretical framework could be built. However, these theoretical developments have not always clearly outlined the processes implicated in the development of normative misperceptions (e.g., Rimal and Real, 2005).

Potentially, a variety of existing theories could be applied to explain how and why normative misperceptions develop. As has been demonstrated extensively in social psychological research, individuals are prone to fundamental attribution error (Gilbert and Malone, 1995), in which we misunderstand the behavior of others by failing to fully acknowledge the possible external causes of their actions. For example, even if we are visiting a bar for the first time in several months we will tend to assume that the other people we see there are regular attendees. Other cognitive biases may be responsible for creating misperceptions, such as false consensus (Ross et al., 1977), in which we erroneously assume others to behave and think in a same way to ourselves (for instance a heavy drinker assuming their level of alcohol consumption to be typical). In addition, pluralistic ignorance may lead people to assume that their behavior and attitudes – such as low to moderate drinking – is atypical, when in fact this is the actual norm (Schroeder and Prentice, 1998). These are just three potential theoretical explanations for why social norm misperceptions develop. The development of a unified testable theory which clearly explains how and why misperceptions of social norms develop, how these misperceptions are associated with future changes in behavior and attitudes, and how interventions should be targeted to change behaviors, represents a significant opportunity for the field.

The lack of a unified theory may explain why studies which are explicitly based on the SNA can be conflated with studies and interventions which generally focus on ‘social norms.’ As discussed earlier, the SNA is built on the assumption that misperceptions of peer norms underlies the engagement in negative health behaviors and reduction in positive behaviors. Whilst many empirical studies have demonstrated an association between perceived norms and a range of health behaviors, many broad ‘social norms’ studies do not measure the impact of *misperceptions* on future behavior (e.g., Pelletier et al., 2014; Robinson et al., 2014). There needs to be a clearer distinction in the literature between SNA-consistent studies focusing on assessing and changing misperceptions of social norms, versus studies generally assessing ‘social norms’ or subjective norms.

### Lack of Clarity on How Social Norms Misperceptions Are Challenged in Interventions

The lack of clarity over the specific social norms feedback featured in interventions and the assessment of changes in perceived norms are limitations of the evidence base. Often published descriptions of interventions are missing important details, such as: how feedback highlights the behaviors or attitudes of the broader social group; which referent groups were described in the feedback; and whether feedback compared between personal behaviors or attitudes with ‘actual’ reported norms. Given the SNA’s assumption that misperceptions of social norms drives behavior (Perkins, 1997; McAlaney et al., 2011), many studies do not discuss whether perceived norms were assessed pre- to post-intervention or how misperceptions of social norms were targeted by the intervention (e.g., Palfai et al., 2014). For example, one study compared the effects of a brief healthy eating information intervention on high school students’ eating behaviors and intentions, including messages on the health benefits associated with eating healthily with an additional descriptive or injunctive normative feedback message (Stok et al., 2014). This study did not report assessing the students’ baseline eating behaviors, or their perceived norms pre- or post-intervention, meaning that the effects of their brief normative feedback on behaviors and intentions at follow-up could be due to pre-existing between group differences in eating behaviors rather than an effect of the normative feedback.

Other studies have simply referred to a ‘correction of misperception’ without clarity on how misperceptions were actually changed by the intervention (e.g., Kypri et al., 2004). In addition, some studies do not specify which normative feedback messages were included in the intervention, which referent groups were included in the feedback, or whether feedback was tailored for key sociodemographic characteristics such as age and sex (e.g., Kypri et al., 2004; Kypri and McAnally, 2005). A greater understanding of how interventions target the discrepancy between individuals’ perceptions of social norms and actual reported norms is needed. Specifically, whether the intervention includes an explicit comparison between participants’ own behaviors and attitudes, their perceptions of perceived norms, and the actual reported norms for their referent group.

To evaluate the effectiveness of SNA interventions in reducing the discrepancy between actual and perceived norms, studies need to assess pre- to post- changes in perceived norms. There are examples of published studies claiming to have changed normative misperceptions without an assessment of participants’ perceptions, or changes in misperceptions, over the course of the intervention (Palfai et al., 2014). To enable a full assessment of the mechanism of change following SNA feedback, studies should assess the implicit pathway to behavior change that the SNA advocates. That is, the mediating role of changes in perceived norms in the relationship between exposure to a social norms intervention with post-intervention outcomes. There also needs to be a greater distinction between studies which explicitly test the SNA versus those that include some form of ‘social norms’ feedback without targeting or measuring changes in normative misperceptions. Improved clarity in how ‘social norms’ are defined and measured across studies is also required in order to enable research into the SNA to be distinguished from the wider field of ‘social norms research’ (for a review of the different definitions of social norms see Chung and Rimal, 2016). Differences in how ‘social norms’ are conceptualized and measured in studies have limited our ability to make cross-study comparisons, pool data for meta-analyses, test the assumptions of the SNA and evaluate the approach as a behavior change strategy.

### Improving the Understanding of How Social Norms Feedback Facilitates Behavior Change

The process underlying how viewing SNA feedback leads to changes in behaviors, attitudes and intentions is still unclear. However, preliminary work has sought to understand how individuals process and react when viewing their SNA feedback (e.g., Marley et al., 2016). One possibility is that SNA-based feedback works because of its ability to surprise the recipient, this surprise then translates into a reassessment of one’s beliefs and perceptions about the social environment, peers’ behaviors and attitudes, leading to changes in normative perceptions and ultimately behavior. However, few interventional studies have explored such processes or participants’ experiences of exposure to SNA feedback, particularly the usability of online or offline computerized normative feedback featured in recent interventions. The work that has been conducted has focused on the general experiences of student participants in SNA interventions (e.g., Neighbors et al., 2009, 2012; Collins et al., 2014), but there remains an opportunity to understand how general population groups and those exposed to SNA feedback in non-university settings react to and comprehend social normative feedback. Repeating an earlier call (McAlaney et al., 2011), there are opportunities for qualitative work to understand user experiences and the processes implicated in challenging and changing normative misperceptions, particularly to inform best practice in future social norms interventions.

### Future Challenges: Changes in Societal Behaviors and Disruptive Technologies

There are a number of challenges facing the future development and use of the SNA. For example, there is some evidence for recent decreases in substance use amongst younger age groups, particularly in the United Kingdom (European Monitoring Centre for Drugs Addiction, 2017; Office for National Statistics, 2017). Such changes in behavior patterns may reflect broader shifts in societal norms. What implications this has for the SNA is not fully clear, but interventions based on the approach may need to adapt messaging toward reinforcing individuals’ more accurate perceptions of the actual low levels of negative behaviors to encourage moderation and avoid highlighting self-other discrepancies in normative feedback. Few SNA-informed studies have focused on reinforcing low or more accurate social normative perceptions amongst participants with low levels of negative health behaviors; although, one study with light- and non-drinking university students indicated the possible protective effects of normative feedback against time-related increases in alcohol consumption (Neighbors et al., 2011). A secondary analysis of four studies indicated that presenting normative feedback for alcohol use does not appear to be associated with a ‘boomerang effect’ for students who drink alcohol below reported normative rates, i.e., these light drinking students did not increase their alcohol consumption to match the reported (higher) norms (Prince et al., 2014). Rather, it seems that such students’ lower normative rates of alcohol consumption were reinforced by SNA feedback. However, it is unclear which factors buffer such students from a possible boomerang effect, for example, it may be that a lack of social identification with ‘typical drinking students’ protects lighter drinkers from adjusting their behaviors to conform to reported ‘typical student’ norms. The potential role of SNA-informed interventions in protecting against increases in negative health-behaviors warrants further investigation, particularly where they may have benefit as an early intervention or harm-prevention strategy.

Recent innovations in technology, particularly the development of mobile app software, presents both challenges and opportunities for the SNA. Such new technologies represent an opportunity for SNA-informed studies and interventions to be delivered to mass audiences in an accessible and user-friendly manner. As discussed earlier, only one study has used social networking media to deliver SNA-informed feedback (Ridout and Campbell, 2014). Given the high use and access to social media and mobile internet-enabled devices amongst younger age groups (Office for National Statistics, 2016), mobile apps and social media could be used to deliver engaging normative feedback and may also assist in countering the problems with attrition associated with many ‘social norms’ interventions (Foxcroft et al., 2015). There is also the need to consider the influence of social networking sites and apps on health-related behaviors and in the development of misperceptions of social norms. For example, research has suggested that viewing sexually suggestive Facebook photographs is associated with higher perceived rates of unprotected sex and sex with strangers amongst university students’ peers compared to viewing non-suggestive photographs (Young and Jordan, 2013). Exposure to alcohol-related content on social networks has been associated with heavier alcohol usage amongst adolescents and young adults (e.g., Hoffman et al., 2017), especially amongst those who placed greater importance on their online social identity and who spend low-to-moderate time using social networking media (Pegg et al., 2018). Experimental research has also reported increases in perceived university student drinking norms after exposure to a fictitious Facebook profile containing alcohol-related comments and photographs of alcohol consumption at social events (Fournier et al., 2013). Other behaviors like hookah and cannabis use are often positively portrayed on online social networks, which could make such low prevalence behaviors appear to be more normal and acceptable than the reality (Groth et al., 2017). Social networking media may be a key and highly accessible source of (mis)information about normative behavior which individuals use to guide their own behavior. Online social networks and other mobile technologies may have the potential to feed social norms misperceptions, and encourage negative health behaviors, as much as be a tool to challenge misperceptions and promote healthy behaviors.

## Recommendations for Good Practice Using the Social Norms Approach

Based on this critical appraisal there are three core recommendations for good practice using the approach. First, there is a significant need for greater clarity in the reporting of interventional studies testing the Social Norms Approach, including those non-SNA ‘social norms’ studies. Greater clarity about the specific social normative feedback included in the intervention arms of studies, examples of the social norms feedback used, and clarity about whether feedback facilitated an explicit comparison between individuals’ normative perceptions versus actual norms is needed to identify those studies which are explicitly based on the SNA. Furthermore, in line with existing guidance (McAlaney et al., 2010), studies should specify what data normative feedback messages were based on, whether that be existing normative data from the target group, broader national norms, or ideally data taken from a baseline survey conducted with the same participants who are later exposed to the intervention. Delivering normative feedback that is relevant to the target population is important to ensure SNA interventions are perceived to be realistic and persuasive by participants, doing so may underline the effectiveness of the SNA as a behavior change intervention.

Second, there is a need for more specific testing of the SNA’s assumptions. At a minimum, interventions should assess participants’ normative perceptions pre- and post-intervention, evaluate whether interventions are associated with changes in perceived norms, and analyze how changes in perceived norms explain changes in behaviors, attitudes and/or intentions. Although some interventional studies have tested and supported the role of this pathway in eliciting behavior change (e.g., Neighbors et al., 2009; LaBrie et al., 2013; Lewis et al., 2014), this should be a core component of the evaluation of future SNA interventions. Intervention studies should be focused on falsifying the approach’s underlying assumptions to determine the SNA’s viability as a behavior change technique.

Third, and building on the previous point, there needs to be a more robust and systematic evaluation of SNA interventions. In addition to assessing changes in normative perceptions and behaviors over the course of an intervention, SNA interventions should also incorporate process evaluations, qualitative data on participants’ experiences of exposure to normative feedback, as well as researcher- and practitioner-led reflections on the challenges associated with delivering SNA-informed feedback. Ensuring a more rigorous evaluation of SNA interventions would facilitate a more nuanced understanding of the challenges and best practices associated with conducting good quality SNA interventions, and improve the understanding of how participants react to, comprehend and process social normative feedback. A minority of studies have reported process evaluations and analyses of participant feedback and experiences of exposure to normative feedback (e.g., Hallett et al., 2009; Neighbors et al., 2012; Marley et al., 2016), but there remains a need for process evaluations to become part of routine practice when evaluating SNA interventions.

## Conclusion

This critical review has appraised the use of the Social Norms Approach as a health behavior and attitude change technique, identified the challenges and opportunities associated with the approach, and outlined some good practice to consider when using the SNA in empirical research and interventions. The Approach’s key strengths remain in its focus on promoting health behaviors in a positive manner through information-based interventions which challenge commonly held normative misperceptions of various health-related behaviors. Whilst some studies have indicated that challenging misperceptions of perceived norms is associated with positive health behavior and attitude change, there are many methodological issues with the assessment of normative misperceptions, the conflation of different forms of ‘social norms’ studies with those explicitly based on the SNA, and in the poor design, reporting and evaluation of many studies. These issues hinder the evaluation of the approach as a behavior change technique and attempts to falsify its underlying assumptions. We call for greater clarity and rigor in the evaluation of the Social Norms Approach to assist in determining its effectiveness in promoting healthy behavior. Ensuring a more robust evaluation of SNA-based interventions, greater methodological rigor, and improved clarity in the reporting of studies which test the assumptions of the approach, will facilitate the evaluation and the advancement of the Social Norms Approach.

## Author Contributions

RD, JM, and BB contribution to the conception of this review. RD wrote the first draft of the review. All authors contributed to the subsequent drafting and rewriting of the review, and read and approved the final version of the review for submission.

## Conflict of Interest Statement

The authors declare that the research was conducted in the absence of any commercial or financial relationships that could be construed as a potential conflict of interest.
